# Argininosuccinate synthetase 1 suppression and arginine restriction inhibit cell migration in gastric cancer cell lines

**DOI:** 10.1038/srep09783

**Published:** 2015-04-30

**Authors:** Yan-Shen Shan, Hui-Ping Hsu, Ming-Derg Lai, Meng-Chi Yen, Wei-Ching Chen, Jung-Hua Fang, Tzu-Yang Weng, Yi-Ling Chen

**Affiliations:** 1 Department of Surgery, National Cheng Kung University Hospital, College of Medicine, National Cheng Kung University, Tainan, Taiwan; 2 Department of Biochemistry and Molecular Biology, College of Medicine, National Cheng Kung University, Tainan, Taiwan; 3 Institute of Basic Medical Sciences, College of Medicine, National Cheng Kung University, Tainan, Taiwan; 4 Department of Emergency Medicine, Kaohsiung Medical University Hospital, Kaohsiung Medical University, Kaohsiung, Taiwan; 5 Laboratory Animal Center, College of Medicine, National Cheng Kung University, Tainan, Taiwan; 6 Department of Senior Citizen Service Management, Chia Nan University of Pharmacy and Science, Tainan, Taiwan

## Abstract

Gastric cancer metastasis remains a major cause of cancer-related deaths. There is an urgent need to develop new therapeutic approaches targeting metastatic gastric cancer. Argininosuccinate synthetase 1 (ASS1) expression is increased in gastric cancer. We detected the protein expression of ASS1 in human gastric cancer cell lines (AGS, NCI-N87, and MKN45) and in murine gastric cancer cell lines (3I and 3IB2). We used vector-mediated short hairpin RNA (shRNA) expression to silence ASS1 expression in the MKN45 and 3IB2 cell lines, and analyzed the effects of this protein on cell migration and metastasis. We demonstrated that ASS1 silencing suppressed cell migration in the MKN45 and 3IB2 cell lines. ASS1 knockdown significantly reduced liver metastasis in mice after the intrasplenic implantation of 3IB2 cancer cell clones. To determine whether arginine restriction may represent a therapeutic approach to treat gastric cancer, the sensitivity of tumor cells to arginine depletion was determined in gastric cancer cells. Arginine depletion significantly inhibited cell migration in the gastric cancer cell line. The silencing of ASS1 expression in MKN45 and 3IB2 gastric cancer cells markedly decreased STAT3 protein expression. In conclusion, our results indicate that the ASS1 protein is required for cell migration in gastric cancer cell lines.

Aberrant cellular metabolism is vital for tumor progression and metastasis^1^. Novel potential therapeutic targets have been identified by analyzing the metabolic enzymes that are active in human gastric cancer tumors and cell lines. Based on previous studies, supplementing the diet with arginine enhances carcinogenesis in the small intestine and colon^2^,^3^. By contrast, deprivation of dietary arginine decreases tumor development and metastasis^4^,^5^. Previous studies have demonstrated that the pro-inflammatory cytokines tumor necrosis factor alpha (TNF-α) and interleukin-1 beta (IL–1β) regulate argininosuccinate synthetase 1 (*ASS1)* in cancer cell lines^6^. However, the biological effect of ASS1 on gastric carcinogenesis/metastasis remains largely unclear.

Elevated levels of ASS1 mRNA have been reported in primary epithelial ovarian, gastric, colorectal, and lung cancers compared with its expression in corresponding normal tissues^6^,^7^,^8^. The upregulation of the ASS1 protein has been implicated in the carcinogenesis of human gastric cancer^6^,^7^,^8^. In an attempt to develop novel therapeutic approaches for metastasis, we hypothesized that ASS1 overexpression may play an important role in metastatic gastric cancer. We determined ASS1 expression in three different human gastric cancer cell lines (AGS, NCI-N87, and MKN45) and in a murine gastric cancer cell line (3IB2) that was originally derived from an orthotopic transplantable gastric cancer in ICR mice^9^,^10^. It has been reported that murine gastric cancer cells serve as a useful experimental model for exploring the biological effects of different pathways associated with metastasis. In this study, we used an RNA interference (RNAi) approach to target ASS1, a key enzyme involved in arginine metabolism, in the MKN45 and 3IB2 cell lines. The study of stable ASS1 knockdown cells indicated that this protein plays an important role in cell migration. However, the suppression of its expression did not influence cell proliferation *in vitro*. Experiments using a 3IB2 murine gastric cancer model further supported the notion that ASS1 is essential for metastasis.

## Results

### ASS1 expression in human gastric cancer cell lines

We first detected the protein expression of ASS1 in three human gastric cancer cell lines. Among the three examined cell lines (MKN45, AGS and NCI-N87 cells), ASS1 protein expression was significantly higher in NCI-N87 and MKN45 cells than in AGS cells (Supplementary Figure S1a). These results confirmed the expression of ASS1 in gastric cancer cell lines.

### Suppressing ASS1 expression did not influence cell proliferation in MKN45 human gastric cancer cells or 3IB2 murine gastric cancer cells

**Figure 1:**
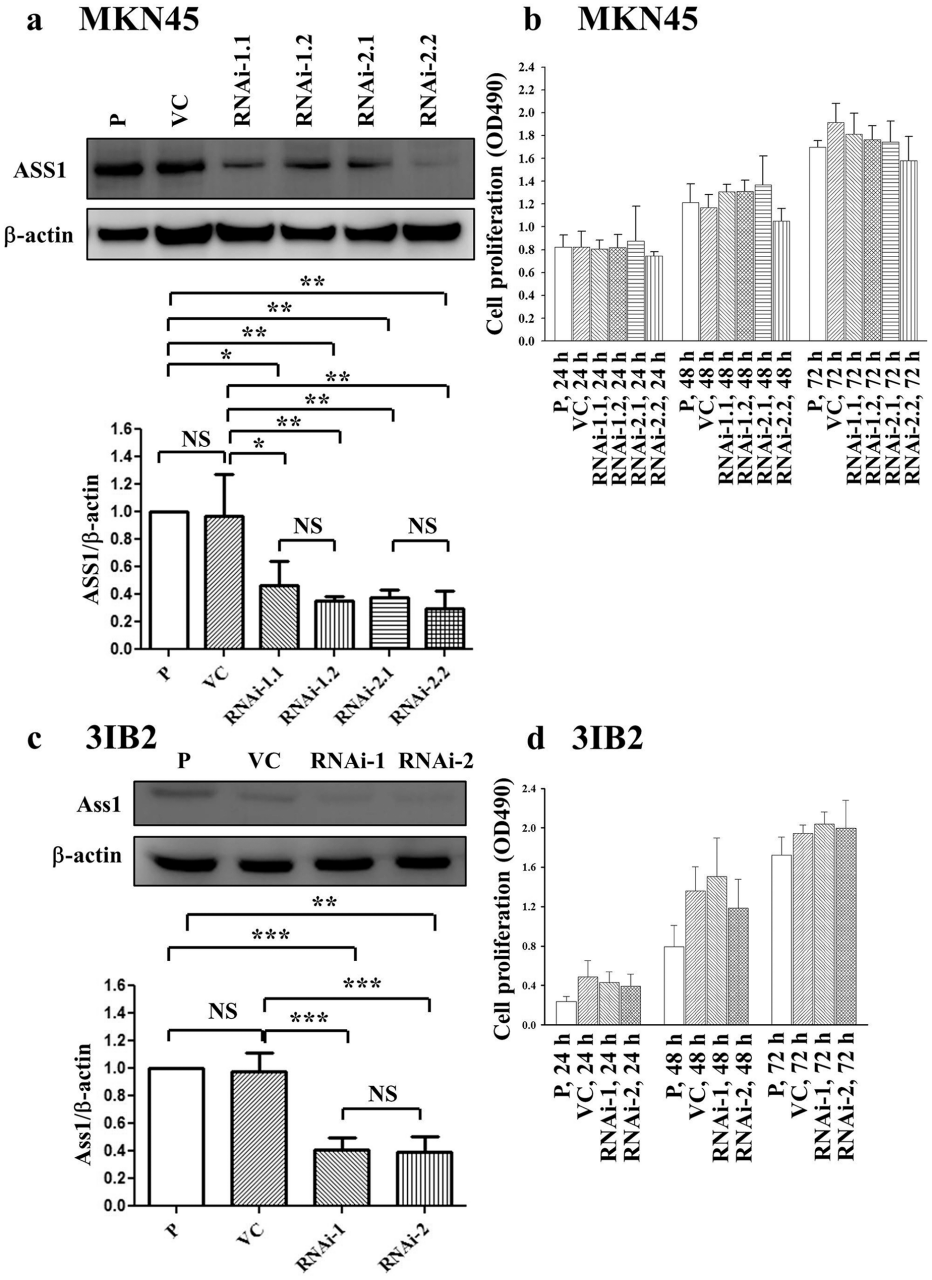
ASS1 silencing in MKN45 and 3IB2 cell clones decreased ASS1 protein expression but did not affect cell proliferation *in vitro*. (a) ASS1 protein expression was determined in human MKN45 cells and ASS1 shRNA stable transfectants. (b) The proliferation of MKN45 cells and ASS1 shRNA stable transfectants was determined at 24, 48, and 72 h. (c) Ass1 expression was determined in murine 3IB2 cells and ASS1 shRNA stable transfectants. (d) The proliferation of 3IB2 cells and ASS1 shRNA stable transfectants was determined at 24, 48, and 72 h. The results of Western blot analysis of protein expression, which were obtained from three independent experiments, are shown in Supplementary Figure S2. The bars represent the mean ± s.d. P: parental cells; VC: vector control; RNAi-1 and RNAi-2: ASS1-specific shRNAs 1 and 2, respectively. NS, not significant, *P < 0.01, **P < 0.001, ***P < 0.0001.

To study the effect of ASS1 on human gastric cancer cells, we used vector-mediated short hairpin RNA (shRNA) to silence ASS1 expression in the MKN45 human gastric cancer cell line. We first constructed a lentiviral vector expressing ASS1 shRNA and infected the MKN45 cell line. To silence ASS1, MKN45 cells were transiently infected with an ASS1 RNAi-1, ASS1 RNAi-2 or control RNAi expression vector. Compared with the vector control and non-silencing RNAi, the ASS1 RNAi-1 and ASS1 RNAi-2 decreased the protein levels of ASS1 (Supplementary Figure S1b). These data suggest that lentivirus-mediated ASS1 RNAi effectively inhibits endogenous ASS1 expression in MKN45 cells. For the study of long-term ASS1 function in gastric cancer, we established four stable cell lines expressing two different RNAi constructs, RNAi-1.1, RNAi-1.2, RNAi-2.1, and RNAi-2.2. ASS1 silencing led to a decrease in the expression of the ASS1 protein in MKN45 cancer cells compared with parental and vector control (VC) cells (Figure 1a and Supplementary Figure S2). The growth rates of the MKN45 cell clones at different intervals were determined by a cell proliferation assay. ASS1 silencing did not affect the proliferation of the MKN45 cells (Figure 1b). These results confirm that ASS1 suppression exerts no significant effect on cell growth in the MKN45 human cancer cell line.

To examine the possible roles of Ass1 in murine gastric cancer cells, we knocked down the expression of Ass1 using shRNA. We used two vector-based shRNAs to silence Ass1 expression, and the resulting cell lines were designated Ass1 RNAi-1 and Ass1 RNAi-2. To confirm the efficacy of the Ass1 shRNAs, we performed Western blotting. Ass1 expression was significantly decreased in the Ass1-suppressed cell clones (transfected with Ass1 RNAi-1 or RNAi-2) according to Western blot analyses (Figure 1c and Supplementary Figure S2). We further determined whether the downregulation of Ass1 affected cell growth in a murine gastric cancer cell line. We found that its silencing did not inhibit the proliferation of the transfectants (Figure 1d). In addition, we examined cell cycle progression in the parental cells and transfectants and did not detect any change in the proportion of cells in the G1 (Supplementary Figure S3a) or S phase (Supplementary Figure S3b). These results reveal that Ass1 suppression does not affect the growth or cell cycle progression of gastric cancer cells. To confirm these results, 3IB2 cells were treated with different concentrations (0.5, 1, 5, or 10 mM) of the Ass1 inhibitor α-methyl-dl-aspartic acid (MDLA)^11^. This treatment exerted no significant effect on cell growth (Supplementary Figure S3c). Thus, Ass1 downregulation or inhibition did not affect gastric cancer cell growth.

### ASS1 suppression attenuated cell migration in human and murine gastric cancer cells

**Figure 2:**
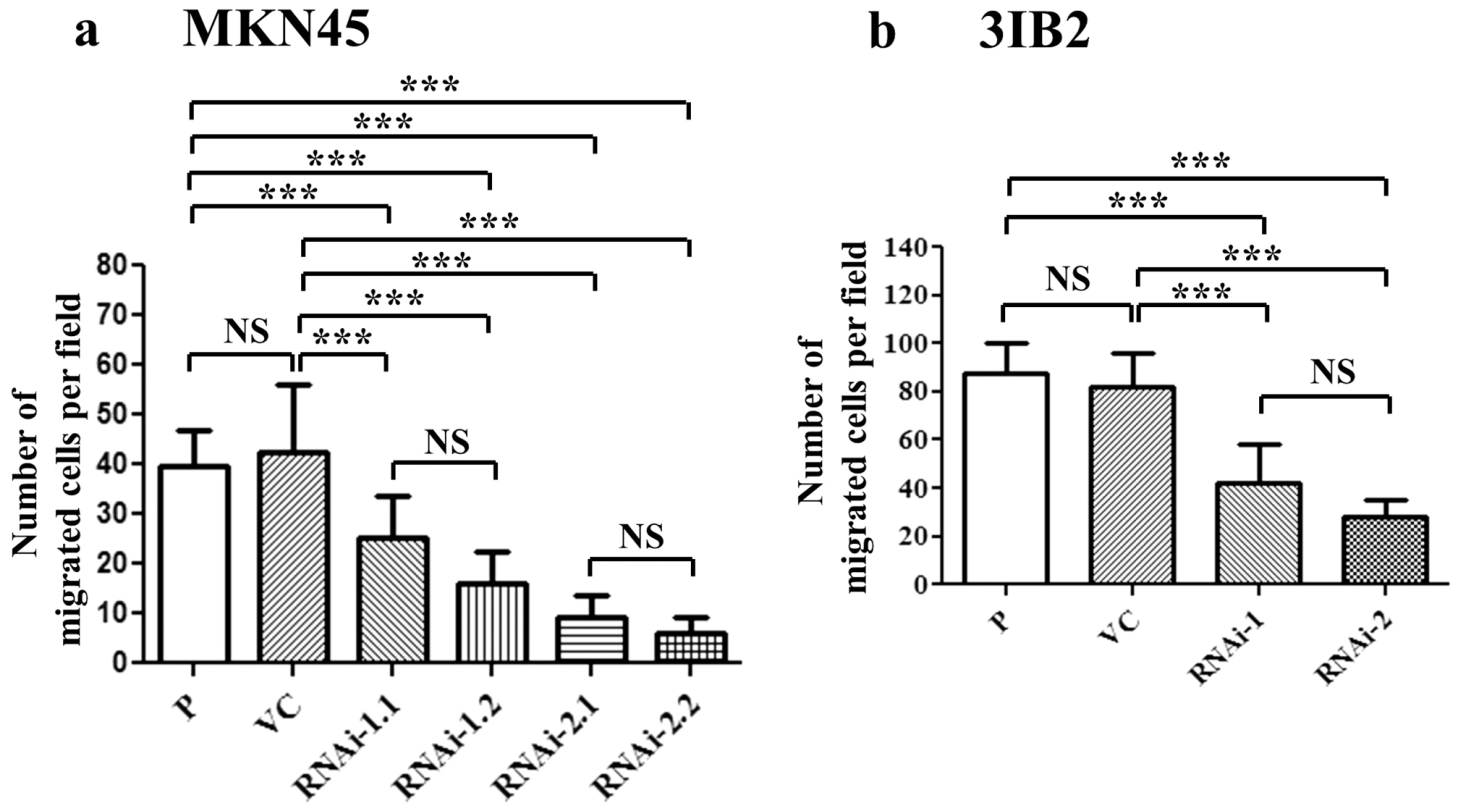
ASS1 silencing in MKN45 and 3IB2 cell clones suppressed cell migration *in vitro*. (a) ASS1-silenced cell clones were examined by the wound-healing assay. The quantitative results of the *in vitro* wound-healing assay at 12 h. (b) Ass1 silencing in the 3IB2 cell clones suppressed cell migration *in vitro*. The quantitative results of the *in vitro* wound-healing assay at 8 h. The data represent the mean ± s.d. of three independent experiments. P: parental cells; VC: vector control; RNAi-1 and RNAi-2: Ass1-specific shRNAs 1 and 2, respectively. NS, not significant, *P < 0.01, **P < 0.001, ***P < 0.0001.

To test the hypothesis that ASS1 plays an important role in tumor cell migration, we determined the changes in the cell motility of MKN45 cell clones *in vitro*. We compared the number of migrating cells to assess the effect of ASS1 on cell motility. ASS1 suppression reduced the number of migrating cells at 12 h, as shown by the wound-healing assay (Figure 2a and Supplementary Figure S4a). The downregulation of ASS1 in MKN45 cells by two different shRNAs resulted in reduced migration. All four stable transfectants displayed similar low migratory abilities. We then determined the effects of Ass1 downregulation on the migration of murine gastric cancer cells. Ass1 suppression reduced the number of migrating cells at 8 h, as demonstrated by the wound-healing assay (Figure 2b and Supplementary Figure S4b). Ass1 RNAi-1- or RNAi-2-transfected 3IB2 cells exhibited a relatively low migration potential. In addition, the motility of the cell clones was assessed by incubating the cells in Boyden chambers for 8 h and using 10% FBS as a chemoattractant. The results revealed that the parental 3IB2 cells and VC cells exhibited relatively high migration potentials. Conversely, Ass1 suppression resulted in a reduction in cell migration, as shown by the Boyden chamber assay (Supplementary Figure S3d).

### Increased Ass1 expression in a metastatic murine gastric cancer cell line

We next investigated whether there is a correlation between Ass1 expression and the migration potential of gastric cancer cells. Ass1 expression was measured in murine 3I and 3IB2 cells. The 3IB2 cell line, which was derived from the 3I murine gastric cancer cell line, displayed a higher metastatic potential than the 3I cell line. The protein expression of Ass1 was elevated in the 3IB2 cell line (Supplementary Figure S1c). We further compared the motility of 3I and 3IB2 cells by a wound-healing assay and found that the 3IB2 cells displayed greater motility than the 3I cells (Supplementary Figure S4c). Therefore, the correlation between metastatic/migration potential and Ass1 expression in these murine gastric cancer cell lines further support an important role of Ass1 in mediating metastasis.

### Effect of ASS1 suppression on tumor metastasis in human gastric cancer cells

**Figure 3:**
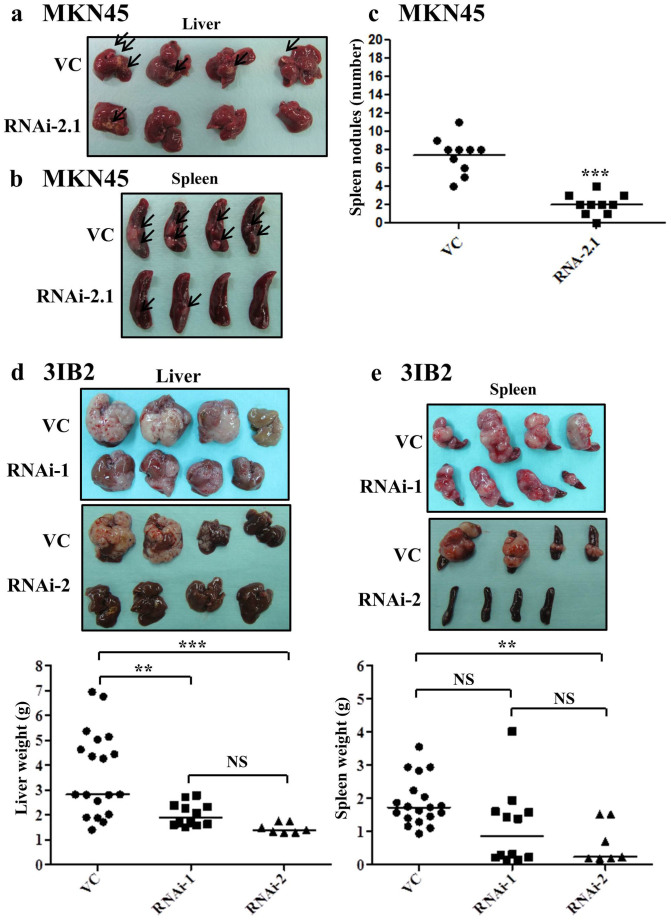
ASS1 silencing in MKN45 and 3IB2 cell clones suppressed tumor metastatic ability *in vivo*. Tumor masses from the liver (a) and spleen (b) were visualized macroscopically after intrasplenic injection of the VC and ASS1-silenced MKN45 cell clones. (c) Spleen nodules in NOD/SCID mice injected with gastric cancer cells on day 14. Tumor masses from the liver (d) and spleen (e) were macroscopically visualized after intrasplenic injection of VC and Ass1-suppressed 3IB2 cell clones. Weights of the liver (d) and spleen (e) of ICR mice injected with gastric cancer cells on day 11. Tumor nodes are indicated by arrows. The results are expressed as the mean ± s.d. of two independent experiments. P: parental cells; VC: vector control; RNAi-1 and RNAi-2: ASS1-specific shRNAs 1 and 2, respectively. NS, not significant, **P < 0.001, ***P < 0.0001.

To examine the hypothesis that ASS1 plays an important role in tumor metastasis, we determined the changes in the metastatic abilities of MKN45 cell clones *in vivo*. MKN45 cell clones metastasized to the liver following injection into the spleen (Figure 3a–c). Macroscopic metastatic nodules indicative of liver metastases were observed in mice injected with VC or ASS1 RNAi-2.1 cell clones (Figure 3a). Then, histopathologic analyses of liver and spleen sections were performed, revealing the presence of tumor nodules after VC or RNAi-2.1 MKN45 cell clone injection (Supplementary Figure S5). The number of nodules in the spleens of the mice injected with VC cells was significantly higher than that of mice injected with ASS1 RNAi-2.1 cells (Figure 3b). The metastatic burden and the number of tumor nodules in the spleen were reduced in the RNAi-2.1 group (Figure 3b&c and Supplementary Figure S5). However, the liver and spleen weights of the MKN45-VC group were not significantly different (1.297 ± 0.375 and 0.080 ± 0.016, respectively) compared with those of the MKN45-RNAi-2-treated group (1.101 ± 0.138 and 0.094 ± 0.048, respectively). These results imply that ASS1 plays a role in the metastasis of human gastric cancer cells.

### Effect of Ass1 suppression on tumor metastasis in murine gastric cancer cells

To further confirm that Ass1 plays an important role in tumor metastasis, we examined the changes in the metastatic abilities of murine gastric cancer cell clones *in vivo*. The 3IB2 murine model is used as an orthotopic intrasplenic implantation model of gastric cancer metastasis to the liver. Parental 3IB2 cells metastasize to the liver when they are injected into the spleen. Following injection of these cells, we observed macroscopic metastatic nodules indicative of the liver metastasis of the cell clones (Figure 3d). The weights of the liver and spleen of the ICR mice injected with VC cells were significantly higher than those of the mice injected with Ass1 RNAi-1 or RNAi-2 cells (Figure 3d&e). Moreover, histopathologic analyses of liver sections were performed, revealing the presence of tumor nodules after VC, RNAi-1, or RNAi-2 3IB2 cell clone injection (Supplementary Figure S6). We used the 3IB2 murine model to demonstrate that the metastatic burden was reduced in the Ass1- transfectants. Taken together, these results indicate that Ass1 plays a significant role in the metastasis of mouse gastric cancer cells.

### Ectopic overexpression of the ASS1 protein in AGS human gastric cancer cells

**Figure 4:**
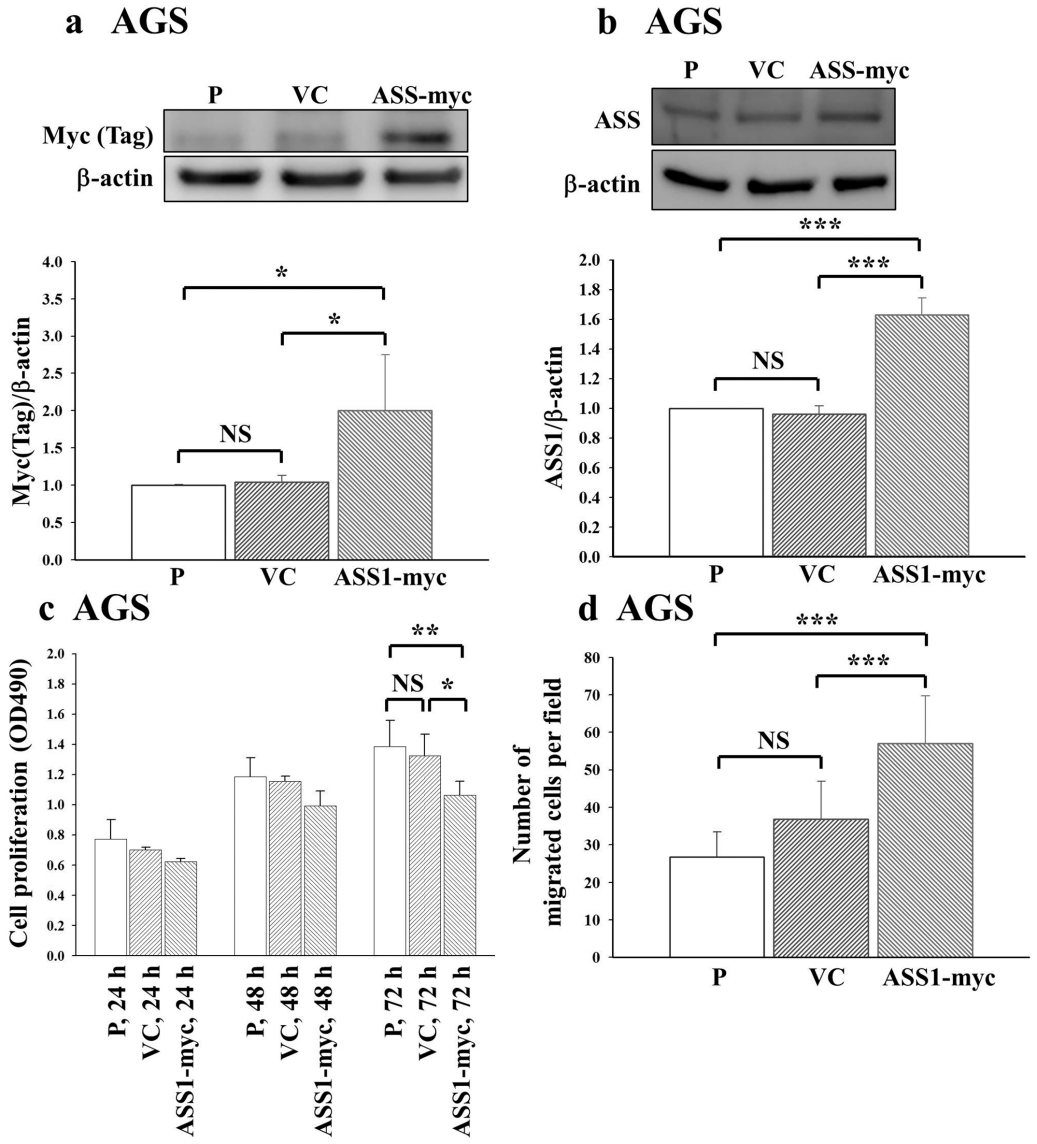
Ectopic expression of the ASS1 protein reduced cell proliferation in an ASS1-overexpressing cell clone. (a) Stable exogenous ASS1 expression was confirmed in AGS cell lines based on the appearance of the expected bands using an anti-Myc antibody. (b) ASS1 protein levels in the cell clones were detected by Western blot analysis. The results of immunoblotting analysis of protein expression are shown in Supplementary Figure S2. (c) ASS1 overexpression in an AGS cell clone inhibited cell proliferation on day 3 *in vitro*. The mean ± s.d. absorbance at 490 nm is shown. (d) An ASS1-overexpressing cell clone was examined by the wound-healing assay. The ASS1-overexpressing cell clone was grown in culture medium containing 10% FCS. All data were obtained from three independent experiments. P: parental cells; VC: vector control; ASS1-myc: ASS1- and myc-overexpressing cells. NS, not significant, *P < 0.05, **P < 0.001, ***P < 0.0001.

To confirm the function of ASS1 in human gastric cancer cell lines, this protein was ectopically expressed in the low-ASS1-expressing AGS human gastric cancer cell line. The establishment of an AGS cell line stably overexpressing ASS1 was confirmed (Figure 4a&b and Supplementary Figure S2). We observed an increase in the ASS1 protein level in the ASS1-myc cell clone (Figure 4a). Elevated expression of ASS1 (> 1.5-fold) was observed in the cell clone ectopically expressing ASS1 (Figure 4b). The ectopic overexpression of *ASS1* weakly inhibited cell growth only on day 3, as shown by cell proliferation assay (Figure 4c). By contrast, its overexpression enhanced the number of migrating cells, as determined by the wound-healing assay (Figure 4d and Supplementary Figure S7a). Taken together, elevated ASS1 protein expression resulted in a relatively high cell migration potential.

### Arginine depletion reduced cell migration in human gastric cancer cells

**Figure 5:**
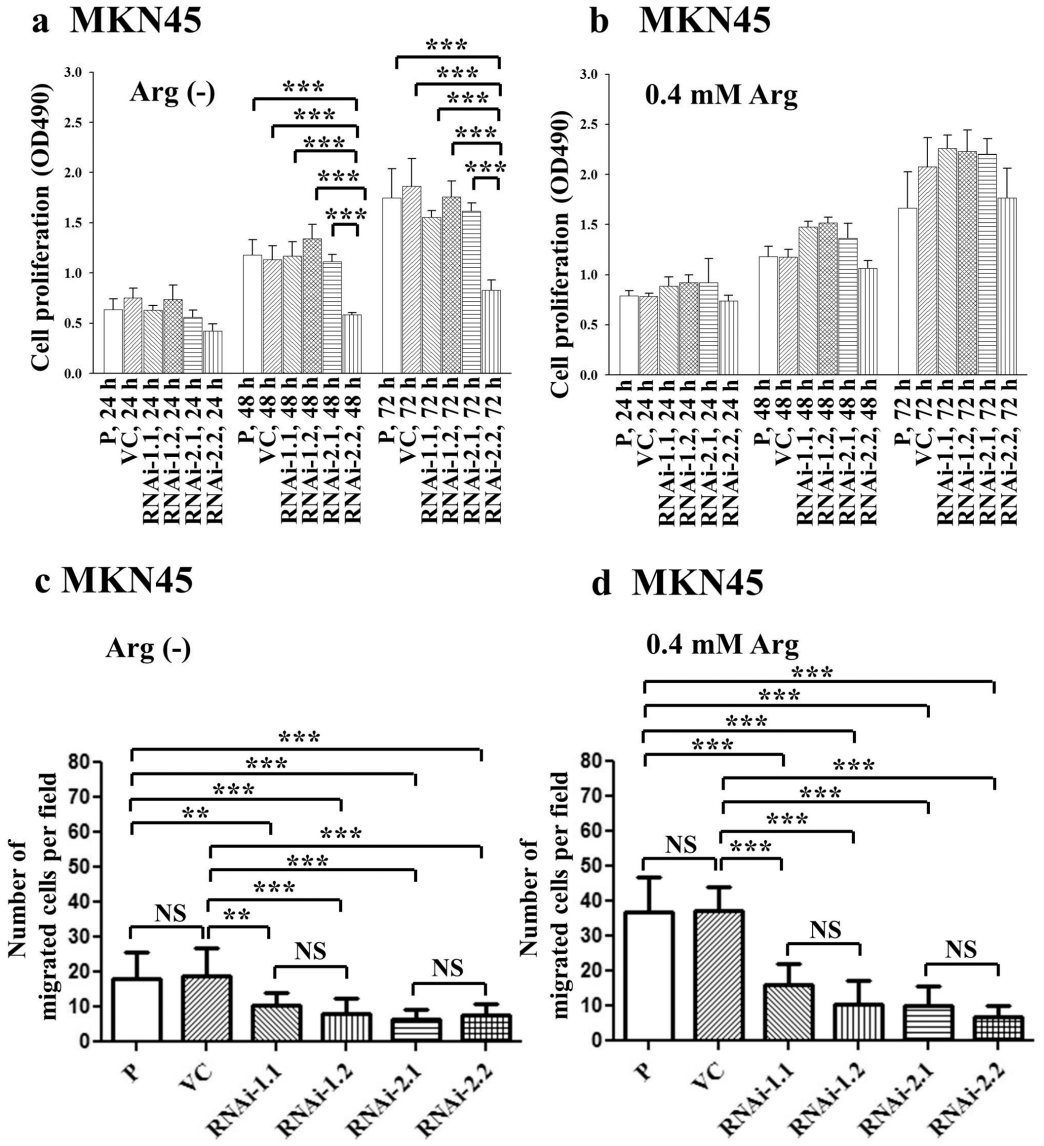
The effects of arginine depletion on the growth and migration of human MKN45 gastric cancer cells. ASS1 knockdown cell clones were grown in culture medium containing 5% FCS. Two different medium conditions were used. Arg (-) indicates arginine-free medium. Cells were cultured for 24, 48, or 72 h. (a&b) Cell proliferation. (c&d) Wound healing. P: parental cells; VC: vector control; RNAi-1 and RNAi-2: ASS1-specific shRNAs 1 and 2, respectively. The results are expressed as the mean ± s.d. of three independent experiments. NS, not significant, **P < 0.001, ***P < 0.0001.

To ascertain whether arginine deprivation may be considered to be a therapeutic approach to treat gastric cancer, we determined whether the expression of ASS1 affects the sensitivity of gastric cancer cells to arginine restriction. Upon arginine withdrawal, the expression of ASS1 did not affect the growth of three of four stable ASS1 knockdown clones. However, the silencing of this protein inhibited cell proliferation in the stable ASS1 RNAi-2.2 MKN45 clone on days 2 and 3 (Figure 5a). In general, the level of ASS1 expression in the gastric cancer cell lines did not affect the growth of these cells in the presence of different arginine concentrations. Upon arginine withdrawal or in the presence of 0.4 mM arginine, ASS1 RNAi-1 and ASS1 RNAi-2 MKN45 cancer cell motility was further inhibited (Figure 5c&d, Supplementary Figure S8). Therefore, the silencing of ASS1 expression and arginine restriction reduce tumor motility in human gastric cancer. Furthermore, we found that ASS1 overexpression did not inhibit cell proliferation either in the complete absence of arginine or in the presence of 0.4 mM arginine (Supplementary Figure S9a&b). ASS1 overexpression also enhanced the motility of AGS cells upon arginine withdrawal or in the presence of 0.4 mM arginine (Supplementary Figures S7b&c and S9c&d).

### The protein expression of ASS1 is associated with that of STAT3 in gastric cancer

**Figure 6:**
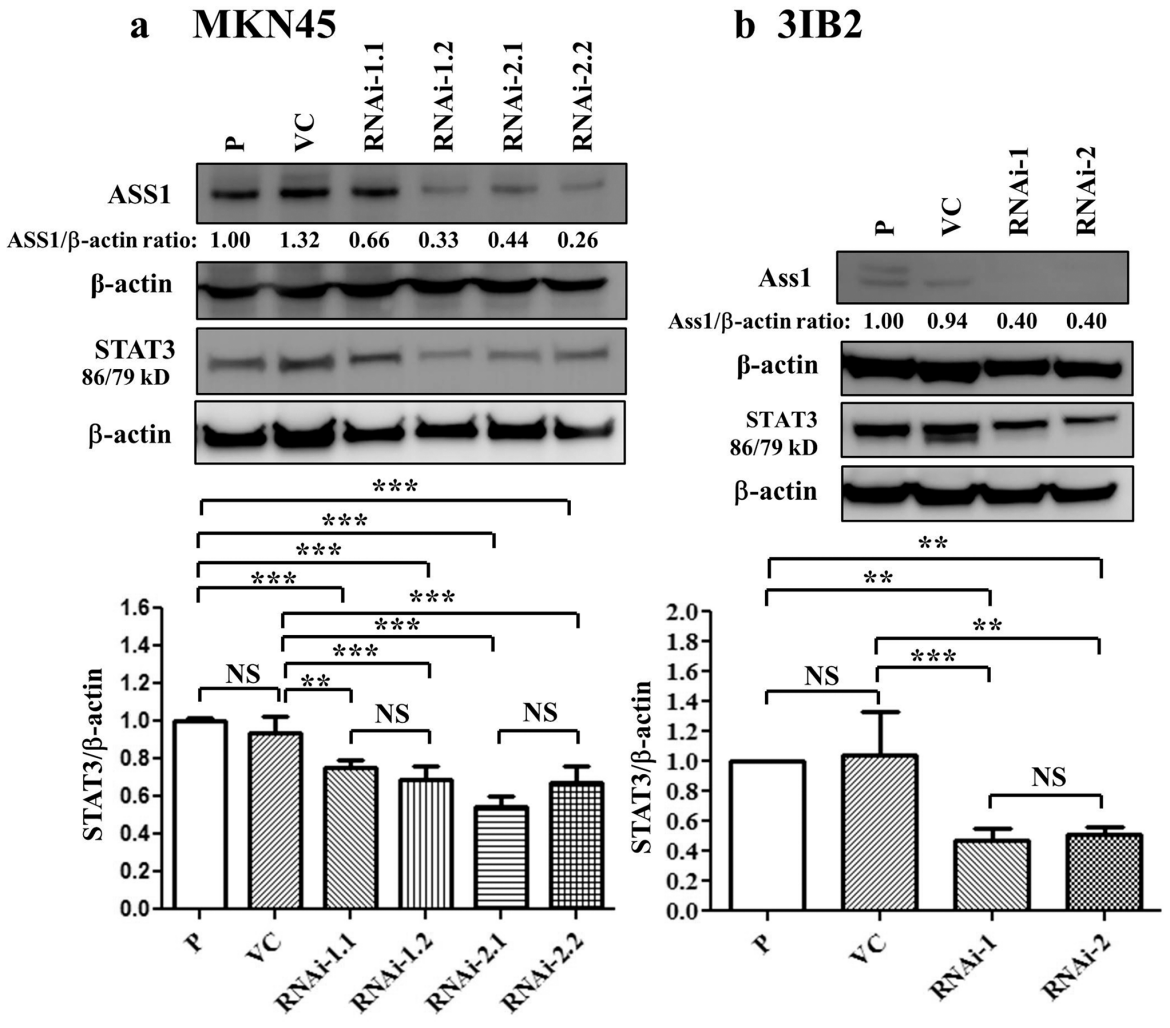
Knockdown of ASS1 expression decreases STAT3 protein expression in gastric cancer cell lines. (a) Western blot analysis of ASS1 and STAT3 in human MKN45 cells and ASS1 stable transfectants. (b) Western blot analysis of ASS1 and STAT3 in murine 3IB2 cells and ASS1 stable transfectants. Ass1-specific shRNA decreased the protein expression of STAT3 in 3IB2 cells. The downregulation of ASS1 decreased STAT3 protein expression in MKN45 and 3IB2 cells. The data represent the mean ± s.d. of three independent experiments. Immunoblotting analysis of protein expression is shown in Supplementary Figure S2. The results were obtained from three independent experiments. STAT3 isoform expression appeared as STAT3α (86 kDa) and STAT3β (79 kDa). P: parental cells; VC: vector control; RNAi-1 and RNAi-2: ASS1-specific shRNAs 1 and 2, respectively. NS, not significant,**P < 0.001, ***P < 0.0001.

Because STAT3 is associated with the metastatic behavior of cancer cells, we hypothesized that ASS1 suppression inhibits STAT3 expression in gastric cancer cells. STAT3 is activated in starved cancer cells^12^, and the expression levels of the STAT3α (86 kDa) and STAT3β (79 kDa) isoforms reflect the biological functioning of STAT3 in different cell types. We observed that AGS, NCI-N87, and MKN45 cells expressed the STAT3 protein (Supplementary Figure S10a). We established MKN45 cells stably transfected with ASS1 shRNA. ASS1 silencing in these cells decreased the protein expression of STAT3 compared with parental and VC cells (Figure 6a and Supplementary Figure S2). To exclude the activation of STAT3 due to long-term culture selection, ASS1 shRNA was delivered transiently to MKN45 cells, resulting in decreased ASS1 expression in the lentivirus-transfected cells (Supplementary Figure S10b). Compared with the vector control and non-silencing RNAi, STAT3 protein expression was decreased by ASS1 RNAi-1 and ASS1 RNAi-2 (Supplementary Figure S10c). These data suggest that ASS1 shRNA effectively inhibits STAT3 protein expression in MKN45 cells. In addition, we established stably transfected ASS1-overexpressing AGS cells. The upregulation of ASS1 enhanced STAT3 protein expression in AGS cancer cells compared with parental and VC cells (Supplementary Figure S11). We also determined the level of STAT3 protein expression in 3IB2 gastric cancer cells and found that 3IB2 cells exhibited higher STAT3 protein levels than 3I cells under serum-deprived conditions (Supplementary Figure S12a). Ass1 and STAT3 expression levels were significantly decreased in the Ass1-suppressed cell clones (Ass1 RNAi-1 and Ass1 RNAi-2) (Figure 6b and Supplementary Figure S12b&c). These results suggest that the silencing of Ass1 expression reduces STAT3 expression in human gastric cancer cell lines. Thus, Ass1 knockdown influences STAT3 expression. Taken together, these results indicate that the protein expression of STAT3 is associated with that of ASS1.

## Discussion

**Figure 7:**
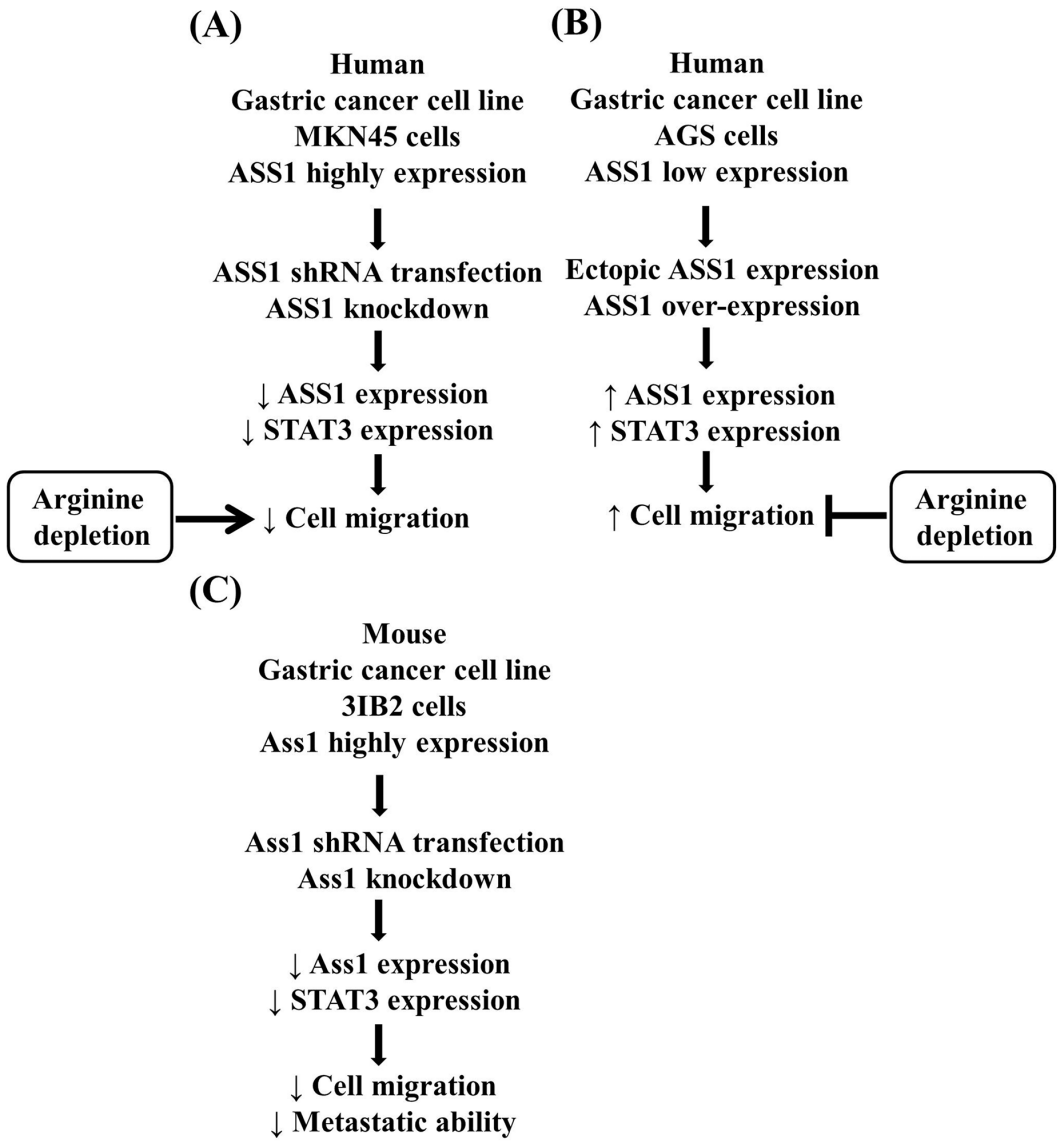
The proposed model depicting the effects of ASS1 on cell migration and metastasis in MKN45, AGS and 3IB2 gastric cancer cell lines. (a) The suppression of ASS1 expression inhibits cell migration in the MKN45 cell line, and this effect is associated with a decrease in STAT3 protein expression. (b) The overexpression of ASS1 increases cell migration in the AGS cell line, and this effect is associated with an increase in STAT3 expression. (c) ASS1 silencing in the 3IB2 mouse gastric cancer cell line inhibits cell migration and tumor metastasis, and these effects are associated with decreased STAT3 expression. ASS1 silencing, ectopic ASS1 expression and the arginine concentration affect cell migration in gastric cancer cell lines.

In the current study, we first determined the expression of ASS1 in human and murine gastric cancer cell lines and found that these cells displayed high protein levels of both ASS1 and STAT3. We further demonstrated the effects of ASS1 on gastric cancer cell migration and metastasis. An outline of the experimental results is illustrated in Figure 7. ASS1 is constitutively expressed in AGS, NCI-N87 and MKN45 cells. The human gastric cancer cell lines NCI-N87 and MKN45 were derived from a metastatic lesion in the liver, and AGS was derived from primary gastric cancer^13^,^14^,^15^,^16^. ASS1 suppression effectively reduced the migration and metastatic potentials of the human MKN45 and murine 3IB2 gastric cancer cell lines *in vitro* and *in vivo*. These data suggest that the elevated expression of ASS1 is associated with the high metastatic potential of gastric cancer.

Forestomach tumors induced by chemical carcinogenesis have generally been shown to be squamous cell carcinomas and not adenocarcinomas^17^,^18^. The mouse gastric cancer cell line 3IB2 was derived from the forestomach and not the glandular stomach^9^. We determined the expression of Ass1 in the 3IB2 mouse gastric cancer cell line, and found that its suppression resulted in decreases in cell migration and tumor metastasis *in vitro* and *in vivo*. We have previously performed immune-histochemical analysis on gastric cancer tissues, and found that ASS1 protein was expressed and localized primarily to the cytoplasm of cancer cells and normal epithelium^19^. We thus hypothesized that the expression of ASS1 in gastric cancer is associated with poor prognosis. In the current study, orthotopic intrasplenic injection of 3IB2 cells was performed to generate a model that mimicked human gastric cancer, displaying a complex metastatic process. When 3IB2 VC cells were transplanted into the spleen, large tumor nodules formed in this organ that further metastasized to the liver. The silencing of ASS1 expression significantly suppressed the metastasis of these cells to the liver and suppressed their tumorigenicity in the spleen. In the present study, we examined the effect of ASS1 on tumor metastasis in only an immune-competent mouse model. Further experiments using other gastric cancer animal models will confirm the effects of this protein on metastasis.

Advanced gastric cancer often becomes resistant to chemotherapy or radiotherapy^20^. Because metastatic disease is frequently incurable, there is an urgent need to develop new therapeutic approaches. To further illustrate the contribution of ASS1 to the migration of gastric cancer cells, we interfered with its functioning using shRNA. ASS1 alteration exerted little effect on the proliferation of various stable transfectants. By contrast, the downregulation of the ASS1 protein and arginine deprivation in gastric cancer cells effectively inhibited cell migration (Figures 5c&d). Moreover, the upregulation of this protein in gastric cancer cells promoted cell migration, independent of the arginine concentration (Supplement Figures S9c&d). The findings of this study implicate arginine as a semi-essential amino acid in the migration of tumor cells. Taken together, the downregulation of ASS1 expression in gastric cancer cells inhibited cell motility and altered cell sensitivity to arginine deprivation. These results suggest that ASS1 expression and the arginine concentration play roles in tumor metastasis.

At present, ASS1 has been found to be involved in tumorigenesis and tumor progression, and some researchers have reported that its expression is decreased in various types of tumors^21^,^22^. Human melanoma and hepatocellular carcinoma (HCC) cells with ASS1 deficiency have been shown to be sensitive to arginine deprivation, such as that induced by treatment with pegylated arginine deiminase (ADI)^23^. However, high ASS1 expression may confer resistance to ADI, suggesting that it only kills cancer cells that lack the expression of this protein^24^,^25^. Similarly, melanoma tumor cells have been reported to display constitutively high levels of ASS1 expression after ADI treatment^26^. Tumors with low ASS1 expression are dependent on extracellular arginine for cell growth and are referred to as arginine auxotrophs^27^,^28^,^29^. In addition, normal cells synthesize arginine intracellularly from ornithine. Arginine synthesis is mediated by ornithine carbamoyl transferase (OCT) during arginine deprivation^30^. OCT is primarily expressed in the liver and the renal/intestinal axis; therefore, other tissues cannot readily convert ornithine to arginine^31^,^32^. Many types of tumor cells die in culture media deficient in arginine, whereas normal cells enter quiescence and survive for long periods of time^33^. Therefore, ASS1 suppression and arginine restriction are promising treatments to hinder metastatic tumor growth. We suggest that ASS1 suppression in gastric cancer provides a survival advantage to gastric cancer patients receiving pegylated arginine deiminase treatment. Further studies are needed to investigate the protein expression of OCT and ASS1 in gastric cancer specimens and their effects on arginine deprivation therapy.

This is the first report to demonstrate that the downregulation of ASS1 expression suppresses STAT3 protein expression and liver metastasis. In a previous study, activated STAT3 has been associated with cell survival and motility^34^. Constitutive STAT3 activation has been reported in human gastric cancer^35^. STAT3 expression has also been correlated with lymph node metastasis in gastric cancer^36^. In addition, this protein causes an increase in cell migration in lung cancer^37^, promotes the angiogenesis of melanoma and hepatocellular cancer in animal models^38^,^39^, and increases cell motility and invasion in ovarian cancer^40^. In the current study, the effect of STAT3 protein expression was shown to be associated with ASS1 expression. Taken together, ASS1 regulates STAT3 signaling and plays an important role in the liver metastasis of gastric cancer. Further investigation is needed to elucidate the mechanism underlying ASS1-mediated STAT3 protein expression during metastasis.

In this report, we have found that decreased ASS1 protein expression or arginine depletion inhibits cancer cell migration. In addition, the level of ASS1 expression in gastric cancer cell lines affects cell motility following arginine withdrawal. Finally, the ASS1 protein promotes the metastatic abilities of tumor cells in experimental models of gastric cancer metastasis *in vivo.* In conclusion, ASS1 is overexpressed in gastric cancer, and the suppression of its expression inhibits tumor migration and metastasis *in vitro* and *in vivo*.

## Methods

### Antibodies

The following antibodies were used in this study: mouse anti-ASS1 (BD Transduction Laboratories, San Jose, CA, USA); mouse anti-myc (BD Biosciences, San Jose, CA); rabbit anti-STAT3 and peroxidase-conjugated goat anti-rabbit IgG (Cell Signaling, Boston, MA, USA); mouse anti-β-actin (GeneTex, Inc., San Antonio, TX, USA); and peroxidase-conjugated sheep anti-mouse IgG (Chemica, San Diego, CA, USA).

### Cell culture

AGS and NCI-N87 human gastric cancer cells were obtained from the Bioresource Collection and Research Center (BCRC, Food Industry Research and Development Institute, Hsinchu, Taiwan), and MKN45 cells were kindly provided by Dr. MD Lai (National Cheng Kung University, Tainan, Taiwan). The AGS, MKN45 and NCI-N87 cell lines were authenticated by DNA (short tandem repeat) profiling at the Bioresource Collection and Research Center in 2013. The cell lines were maintained in Dulbecco's modified Eagle's medium (DMEM) or RPMI1640 medium (Gibco, Life Technologies, Grand Island, NY) containing 10% FBS (Gibco), 100 U/mL penicillin, and 100 µg/mL streptomycin.

To establish a highly tumorigenic stomach cancer cell line (3IB2) displaying properties of putative cancer stem cells, we orthotopically implanted a relatively low number of 3I cells into syngeneic ICR mice^9^. Briefly, 500 3I cells were orthotopically implanted into these mice, and the resultant tumors were isolated and cultured *in vitro* to establish the 3IB2 cell line. Fresh tumor tissues were sliced into small pieces and incubated in digestion buffer containing 0.05% trypsin and 0.02% EDTA. This step was followed by incubation for 30 min at 37°C in a water bath. To obtain clones derived from a single cell, a limiting dilution technique was performed using a mixed population of tumor cells. In our preliminary study, the protein expression of ASS1 was found to be increased in these cells. 3IB2 cells were maintained in high-glucose DMEM supplemented with 10% FBS (Gibco, Life Technologies, Grand Island, NY, USA), 100 U/mL penicillin, and 100 µg/mL streptomycin. The 3IB2 cell lines have now been subcultured for more than 2 years without any apparent phenotypic changes.

### Lentivirus production, RNA interference, transfection and stable cell line generation

For shRNA-mediated silencing, shRNAs targeting ASS1 were constructed in pLKO.1 plasmids obtained from the National RNAi Core Facility (Academia Sinica, Taipei, Taiwan). The target sequences for human ASS1 were 5′-GCCTGAATTCTACAACCGGTT-3′ (RNAi-1) and 5′-CTCAGGCTGAAGGAATATCAT-3′ (RNAi-2), and those for murine Ass1 were 5′-GTCTCCACTTTCACTCTACAA-3′ (RNAi-1) and 5′-CTCCCAGGCTTCAGCATTAAT-3′ (RNAi-2). Lentiviral shRNA clones targeting human ASS1 or a control sequence (PLKO.1 and luciferase non-silencing shRNA) were purchased from the National RNAi Core Facility (Academia Sinica, Taiwan; http://rnai.genmed.sinica.edu.tw). To construct lentiviral particles expressing shRNA, an shRNA plasmid, the packaging plasmid pSPAX2 and the envelope plasmid pM2DG were cotransfected into HEK293T cells using TurboFect transfection reagent (Fermentas*,* Glen Burnie, MD, USA). After 24 h of transfection, the medium was replaced with DMEM containing 10% FBS and 1% BSA. The viral particles in the culture medium were harvested 48 h later and stored at −80°C until use. For ASS1 knockdown, MKN45 cells were plated on 10 cm plates and incubated overnight. They were then infected with shASS1 lentivirus in the presence of Polybrene (Sigma) at a final concentration of 8 μg/mL. The cells were incubated with the virus for 24 h prior to the replacement of the medium with selection medium containing puromycin (1 μg/mL). After incubation for 48 h, total cell lysates were collected. To evaluate transient expression, 3 different transfections were subsequently analyzed. The data represent three independent experiments using different batches of plasmid DNA and cell lines. To monitor the efficacy of ASS1 silencing, the expression of this protein was analyzed in transiently transfected cells by Western blotting.

Cells were transfected with vectors containing shRNA corresponding to the target sequences using the transfection reagent Lipofectamine 2000 (Invitrogen, Life Technologies, Darmstadt, Germany). Single cell clones of the transfectants were selected using the limiting dilution method. We used two different ASS1 shRNAs to establish stably ASS1 knockdown clones of MKN45 and 3IB2 cells via puromycin (1 µg/mL) (Sigma) selection. Two individual clones were obtained from MKN45 cells for each shRNA that were termed RNAi-1.1, RNAi-1.2, RNAi-2.1 and RNAi-2.2. For the 3IB2 cell line, only one clone was selected for each shRNA, and they were termed RNAi-1 and RNAi-2. The selected stable clones were maintained in complete medium containing puromycin. For the stable transfectants, three seedings were performed. To monitor the efficacy of ASS1 silencing, ASS expression in the stable transfectants was analyzed by Western blotting.

### Western blot analysis

Total cell lysates were prepared and analyzed by SDS-PAGE as previously described^9^. For quantification, the bands were measured using an AlphaImager 2200 system (Alpha Innotech, San Leandro, CA, USA) and were normalized to the band density of β-actin. ASS1 expression was quantified and expressed as the ASS1 to β-actin ratio. These experiments were repeated using three independent batches of cell clones or cell lysates. The quantitative data are presented as the values relative to those in the control cells. For more detailed information, please see Supporting Information.

### Cell proliferation assay

3IB2 (2 × 10^3^ cells/well), MKN45 (5 × 10^3^ cells/well) and AGS (5 × 10^3^ cells/well) cells were seeded in triplicate in 96-well plates and incubated at 37°C in 5% CO_2_. The numbers of viable cells were measured using the CellTiter 96 Aqueous One Solution cell proliferation assay (Promega, Madison, WI, USA) according to the manufacturer's instructions. To evaluate cell growth after ASS1 inhibition, MDLA (Sigma-Aldrich, St. Louis, MO, USA) was added at a final concentration of 0 (PBS control), 0.5, 1, 5, or 10 mM for each group^11^. For more detailed information, please see Supporting Information.

### Cell cycle analysis by flow cytometry

Tumor cells were analyzed for changes in the cell cycle status via propidium iodide analysis as previously described^9^. For more detailed information, please see Supporting Information.

### Mice and ethics statement

Six-to-eight-week-old ICR and non-obese diabetic/severe combined immunodeficient (NOD/SCID) mice were purchased from the Laboratory Animal Center of National Cheng Kung University (Tainan, Taiwan) and were maintained under specific pathogen-free conditions. The animal experiments were approved by the Institutional Animal Care and Use Committee of National Cheng Kung University (approval no. NCKU-IACUC-100-179). The methods were performed in accordance with the approved guidelines.

### Experimental metastatic model of gastric carcinoma

The metastatic abilities of the MKN45 and 3IB2 cell clones *in vivo* were evaluated using a hepatic metastasis model, in which 1 × 10^6^ tumor cells in 0.05 mL of PBS were injected intrasplenically as previously described^9^. For more detailed information, please see Supporting Information.

### Wound-healing assay

To evaluate the cell motility of the MKN45, AGS, 3I and 3IB2 cell clones, *in vitro* wound-healing assay was performed^41^. MKN45 (70 μL of 1 × 10^6^ cells per mL), AGS (70 μL of 5 × 10^5^ cells per mL)^42^, 3I or 3IB2 cells (70 μL of 2 × 10^5^ cells per mL) were seeded in an ibidi culture insert (Applied BioPhysics, Inc., Martinsried, Germany) above a 6-well plate. After incubation overnight, the cell culture insert was carefully removed to form a cell-free gap between the attached cells. The incubation time for the wound-healing assay depended on the tumor cells used. Cell motility into this defined wound was observed and analyzed. Six fields were randomly selected, and the number of migrated cells was counted.

### Cell migration assay

Cell migration was evaluated by incubating 3IB2 cell clones in modified Boyden chambers (NeuroProbe, Inc., Gaithersburg, MD, USA) for 8 h as previously described^9^. For more detailed information, please see Supporting Information.

### Ectopic ASS expression

Myc-DDK-tagged ASS1 (RC223189) was purchased from OriGene Technologies Inc. (Rockville, MD). Human ASS1 cDNA was cloned into pCMV6 to produce ASS1 pCMV6. AGS cells were transfected with empty vector or ASS1 pCMV6 using the transfection reagent Lipofectamine 2000. For more detailed information, please see Supporting Information.

### Arginine depletion experiments

Medium containing 0.4 mM arginine (Sigma-Aldrich, St. Louis, MO, USA) and arginine-free medium (Gibco) were supplemented with 5% dialyzed FCS (> 10 kDa; Gibco)^43^,^44^,^45^. For cell growth assays, 2000 cells per well were seeded in quadruplicate in 96-well plates in culture medium containing 5% dialyzed FCS and either no arginine or 0.4 mM arginine.

### Statistical analysis

GraphPad Prism (version 4.00 for Windows; GraphPad Software, San Diego, CA, USA; www.graphpad.com) was used for analyses. The data are expressed as the mean ± standard deviation (s.d.). Statistical analyses were performed using Student's *t*-test or one-way ANOVA followed by Tukey's test. Statistical analyses between two groups were performed using Student's *t*-test. One-way ANOVA was used for multiple group comparisons.

## Author Contributions

The contributions of each author to this study were as follows: Y.L.C. performed most of the experiments, prepared Figures 1–7, and wrote the main text. Y.S.S. and H.P.H. contributed to the experimental design, performed the animal experiments and prepared Figure 3 and Supplementary Figures S5 and S6. The experiments were supported by Y.S.S. and H.P.H. M.D.L. and M.C.Y. contributed to the experimental design and edited the manuscript. W.C.C., J.H.F. and T.Y.W. assisted with the cell culture experiments. Y.S.S. and H.P.H. equally contributed to this paper. All authors reviewed the manuscript.

## Supplementary Material

Supplementary Information Supporting Information
