# Supplementary material for: Argininosuccinate synthetase 1 suppression and arginine restriction inhibit cell migration in gastric cancer cell lines
Source: Sci Rep. 2015 Apr 30;5:9783. doi: 10.1038/srep09783 (PMC4415574; doi:10.1038/srep09783)
Supplement: Supplementary Information — Supporting Information [file srep09783-s1.pdf]

Argininosuccinate synthetase 1 suppression and arginine restriction inhibit cell migration in gastric cancer cell lines

Yan-Shen Shan[1†], Hui-Ping Hsu[1†], Ming-Derg Lai[2,3], Meng-Chi Yen[2,4], Wei-Ching Chen[2,3], Jung-Hua Fang[5], Tzu-Yang Weng[2,3], Yi-Ling Chen[6*]

[1]Department of Surgery, National Cheng Kung University Hospital, College of Medicine, National Cheng Kung University, Tainan, Taiwan

[2]Department of Biochemistry and Molecular Biology, College of Medicine, National Cheng Kung University, Tainan, Taiwan

[3]Institute of Basic Medical Sciences, College of Medicine, National Cheng Kung University, Tainan, Taiwan

[4]Department of Emergency Medicine, Kaohsiung Medical University Hospital, Kaohsiung Medical University, Kaohsiung, Taiwan

[5]Laboratory Animal Center, College of Medicine, National Cheng Kung University, Tainan, Taiwan

[6]Department of Senior Citizen Service Management, Chia Nan University of Pharmacy and Science, Tainan, Taiwan

[†]YS Shan and HP Hsu contributed equally to this work.

*__Correspondence:__ Yi-Ling Chen, PhD

60 Erh-Jen Road, Sec 1, Jen-Te, Tainan 71710, Taiwan (ROC)

Tel.: 886-6-2664911, ext. 3716; Fax: 886-6-2664931; Email: s5887110@nckualumni.org.tw

Western blot analysis

Total cell lysates were prepared and analyzed by SDS-PAGE as previously described[1]. Tumor cells were washed twice with PBS and lysed with ice-cold RIPA buffer (20 mM Tris-HCl, pH 7.5, 1 mM EDTA, 150 mM NaCl, 1% NP-40 and 1% SDS) containing one protease inhibitor tablet (cOmplete, Mini, EDTA-free Protease Inhibitor Cocktail, Roche; Mannheim, Germany). The tumor cells were harvested using a cell scraper and collected in an Eppendorf tube for 20 min at 4°C. The sample was centrifuged for 10 min at 13000 RPM at 4°C, and the supernatant was transferred to an Eppendorf tube. The total protein concentrations in the cell lysates were quantitatively assessed using the Bio-Rad Bradford assay (Mississauga, ON). Immunodetection was performed using an HRP-based SuperSignal Chemiluminescent Substrate (Pierce, Rockford, IL, USA). For quantification, the bands were measured using an AlphaImager 2200 system (Alpha Innotech, San Leandro, CA, USA) and normalized to the band density of β-actin. Argininosuccinate synthetase 1 (ASS1) expression was quantified and expressed as the ASS1 to β-actin ratio. These experiments were repeated using three independent batches of cell clones or cell lysates. The quantitative data are presented as values relative to those in the control cells.

Cell proliferation assay

3IB2 (2×10^3 cells/well), MKN45 (5×10^3 cells/well) and AGS cells (5×10^3 cells/well) were

seeded in triplicate in 96-well plates and incubated at 37°C in 5% CO_2. At daily intervals (at 24, 48, and 72 h), the number of viable cells was measured using the CellTiter 96 Aqueous One Solution cell proliferation assay (Promega, Madison, WI, USA) according to the manufacturer's instructions. Then, 20 μl of CellTiter One Solution was added to each well. After incubation for 1 h, the absorbance at 490 nm was recorded using an ELISA plate reader. To evaluate cell growth after ASS1 inhibition, α-methyl-DL-aspartic acid (MDLA; Sigma-Aldrich, St. Louis, MO, USA) was added at a final concentration of 0 (PBS control), 0.5, 1, 5, or 10 mM for each group[2].

Cell cycle analysis by flow cytometry

Tumor cells were analyzed for changes in cell cycle status via propidium iodide analysis as previously described[1]. Tumor cells (1×10^6 cells/well) were cultured in 6-well plates for 48 h. Analysis was performed using a FACScan instrument (BD Biosciences, San Jose, CA, USA).

Experimental metastatic model of gastric carcinoma

The metastatic abilities of MKN45 and 3IB2 cell clones *in vivo* were evaluated using a hepatic metastasis model, in which 1×10^6 tumor cells in 0.05 mL of PBS were intrasplenically injected as previously described[1]. Fourteen days after MKN45 cell clone injection, the NOD/SCID mice were sacrificed, and liver metastases were examined

macroscopically and histologically. Eleven days after 3IB2 cell clone injection, the ICR mice were sacrificed, and liver metastases were examined macroscopically and histologically. Formalin-fixed paraffin-embedded sections of the liver and spleen were used for hematoxylin and eosin (H&E) staining. Each animal experiment was performed at least twice.

Ectopic ASS expression

Myc-DDK-tagged ASS1 (RC223189) was purchased from OriGene Technologies Inc. (Rockville, MD). Human ASS1 cDNA was cloned into pCMV6 to produce ASS1 pCMV6. AGS cells were transfected with an empty vector or ASS1 pCMV6 using the transfection reagent Lipofectamine 2000. The stable transfectants were selected using G418 (neomycin sulfate, 300 μg/mL). After limiting dilution analysis, cell clones were selected and maintained for further studies using G418. We established vector control (VC) and ASS1-overexpressing (ASS1-myc) clones. The cell lines were maintained in complete medium containing G418. To monitor the efficacy of ASS1 overexpression, the expression of this protein in the stable transfectants was analyzed by Western blotting.

Cell migration assay

Cell migration was evaluated by culturing 3IB2 cell clones in modified Boyden chambers (NeuroProbe, Inc., Gaithersburg, MD, USA) for 8 h as previously described[1]. To determine

cell migration, 2.5×10^4 cells in serum-free medium were added to the upper chamber, and either 10% FCS-supplemented or serum-free medium was added to the bottom chamber as the chemoattractant. The stained cells were counted in 6 randomly selected fields using a microscope at 100x magnification. Each experiment was performed in triplicate and was repeated three times.

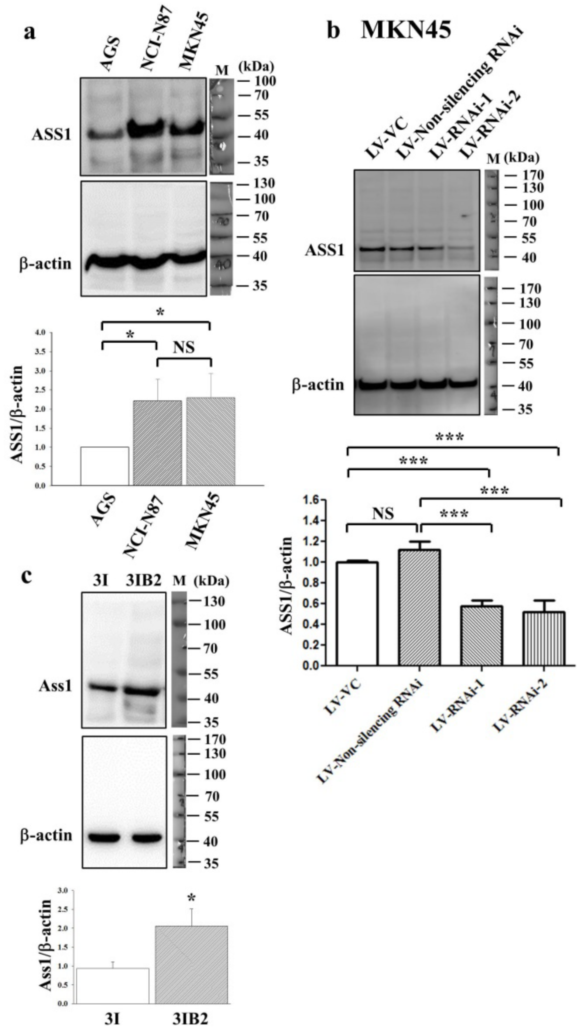

Supplementary Figure S1. (a) The protein expression of ASS1 was determined in AGS, NCI-N87, and MKN45 cells. (b) Confirmation of the knockdown of ASS1 expression in MKN45 cells via lentivirus-mediated RNA interference. ASS1 protein expression in lentivirus-infected cells was measured by Western blotting. (c) The pattern of Ass1 protein expression in 3I and 3IB2 mouse gastric cancer cell lines. The bars represent the mean ± s.d. The results were obtained from three independent experiments. LV: lentivirus; P: parental cells; VC: vector control; RNAi-1 and RNAi-2: ASS1-specific shRNAs 1 and 2. NS, not significant, *P < 0.05, **P < 0.001, ***P < 0.0001.

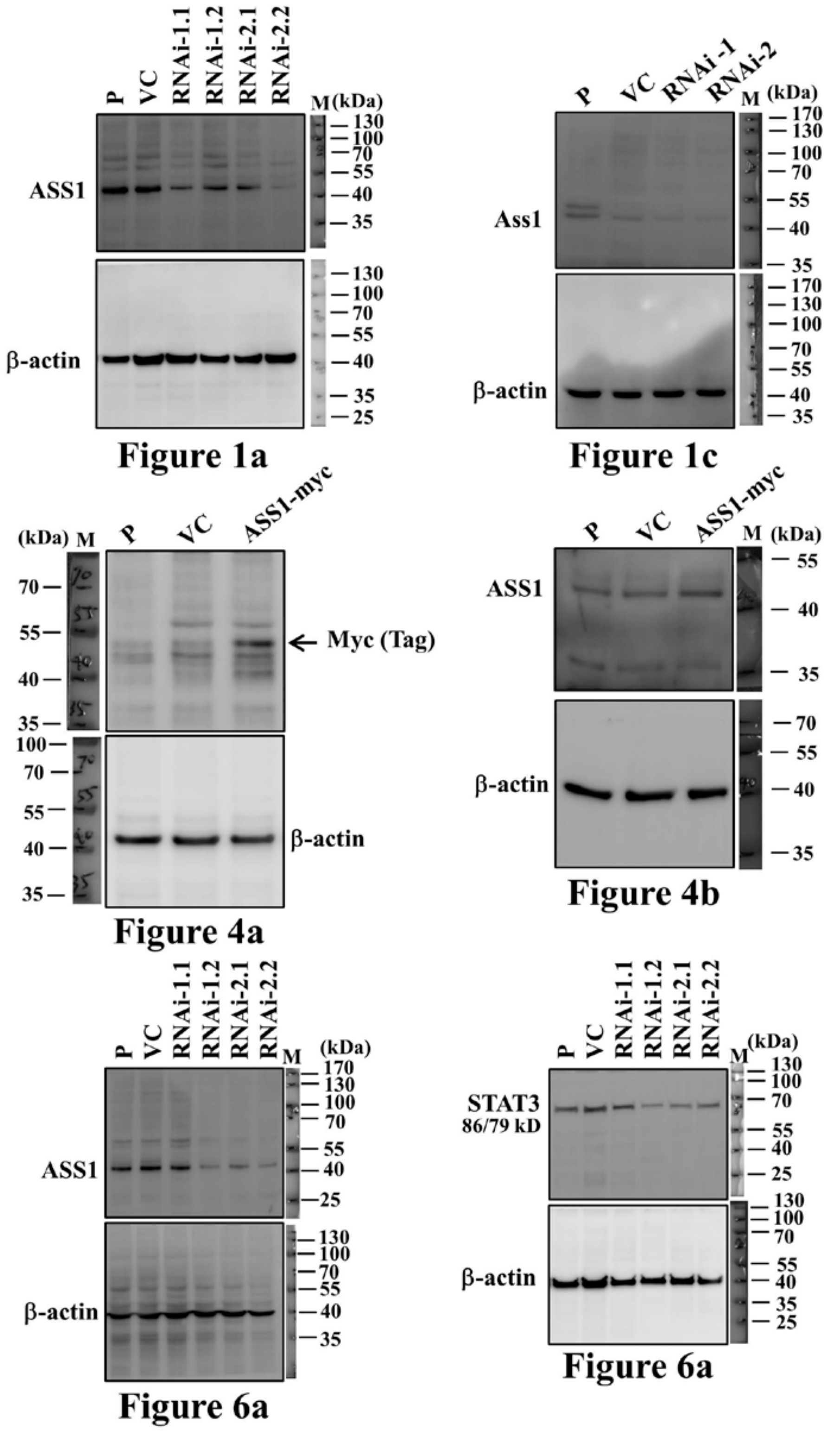

Supplementary Figure S2. Cell lysates were immunoblotted using anti-ASS1, anti-STAT3 and anti-β-actin antibodies. These cropped blots are shown in Fig. 1a, Fig. 1c, Fig. 4a, Fig. 4b and Fig. 6a.

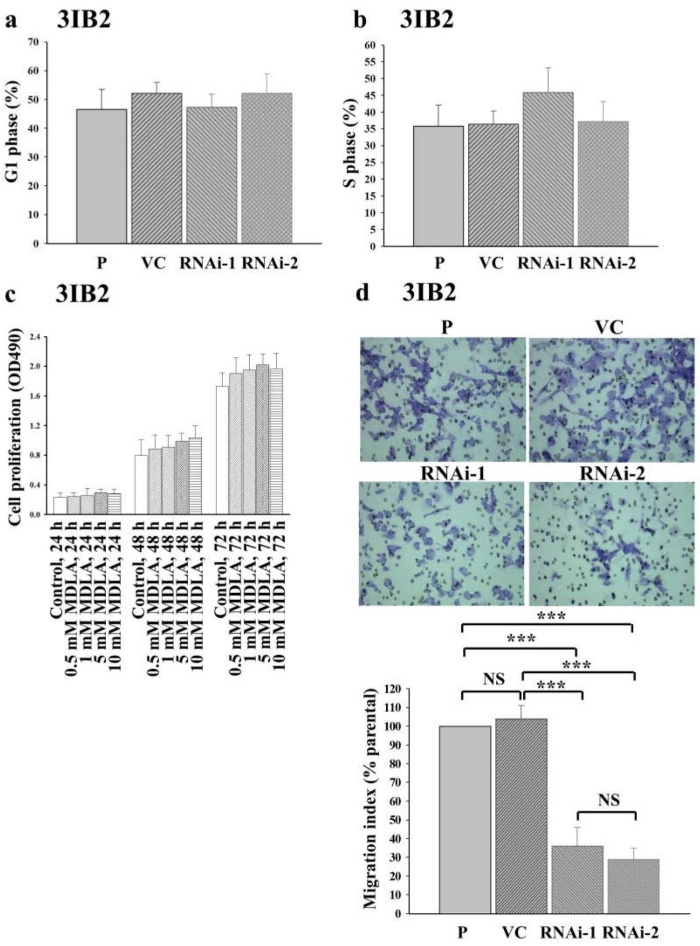

Supplementary Figure S3. Parental, VC, RNAi-1, and RNAi-2 cells were collected, and the percentage of cells in the G1 (**a**) or S (b) phase was analyzed by flow cytometry. The mean ± s.d. is presented as the percentage of cells in the indicated cell cycle phase determined from three independent experiments. (**c**) 3IB2 cells were treated with different concentrations of MDLA (0.5, 1, 5, or 10 mM) for 24, 48, or 72 h. (d) The motility of 3IB2 cells was determined in response to 10% FBS by a Boyden chamber assay followed by light microscopy. A polycarbonate filter with 8-µm pores is visible in the background. Magnification: 100x. The bars represent the mean ± s.d. The data were obtained from three independent experiments.

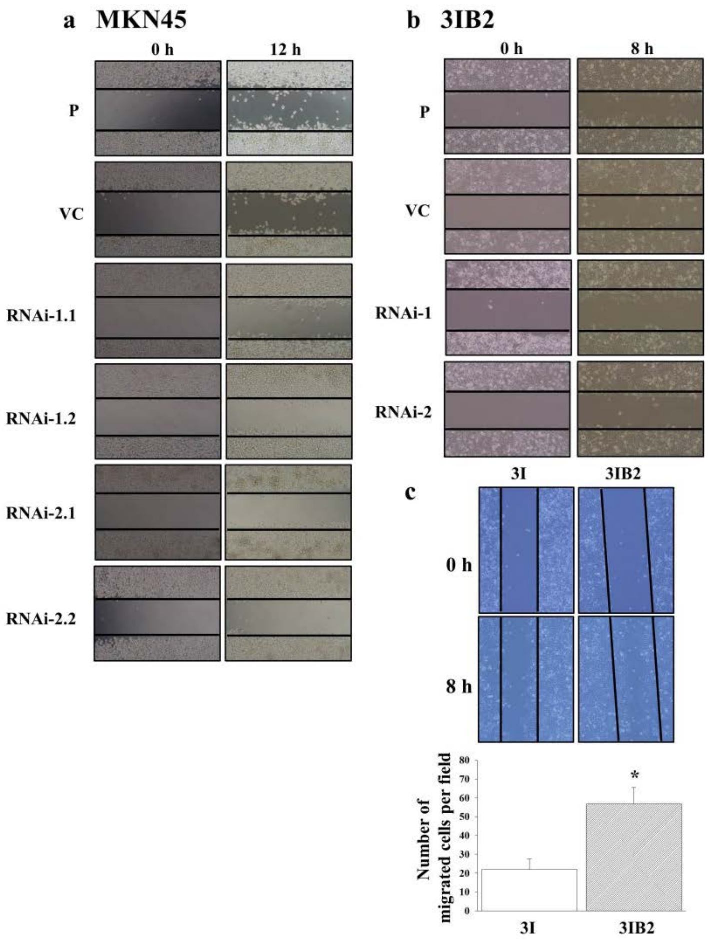

Supplementary Figure S4. The level of ASS1 expression in gastric cancer cell lines affected cell migration. (a) ASS1 silencing in MKN45 cell clones suppressed cell migration *in vitro*. Representative micrographs of one of the individual experiments at time points 0 and 12 h are shown. Cell motility toward the gap area was photographed after 12 h. (b) Ass1 silencing in the 3IB2 cell clones suppressed cell migration *in vitro*. (c) The pattern of cell migration in 3I and 3IB2 mouse gastric cancer cell lines. Representative micrographs of one of the individual experiments at the time points 0 and 8 h are shown. Cell motility toward the gap area was photographed after 8 h. The bars represent the mean ± s.d. The quantitative results of the *in vitro* wound-healing assay at 8 h. *P < 0.05 compared with the 3I cells.

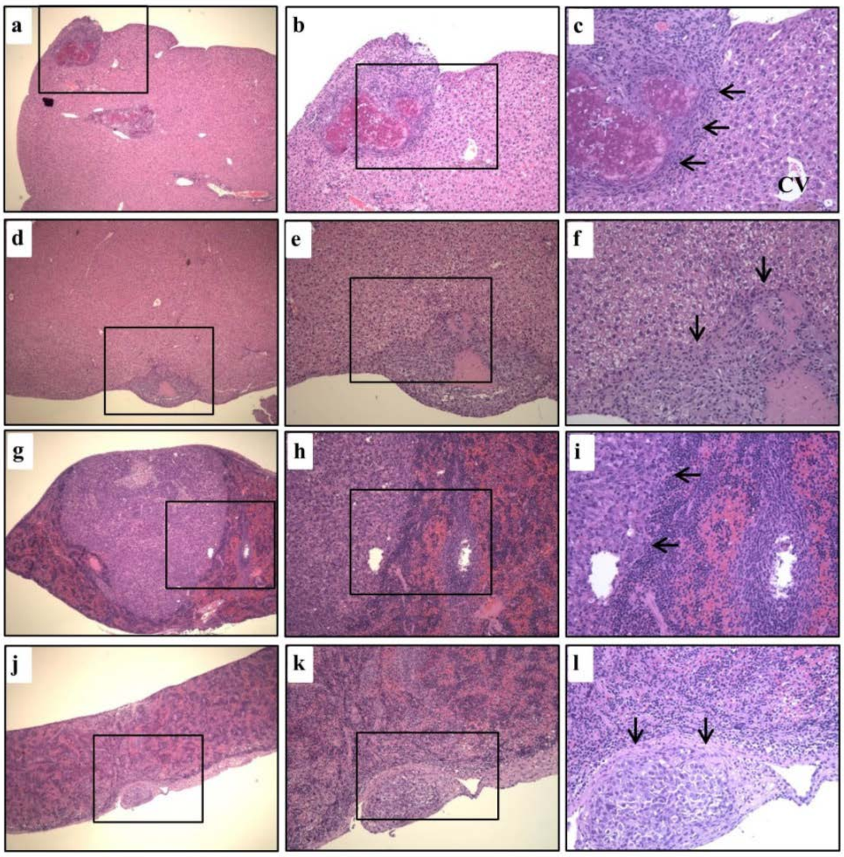

Supplementary Figure S5. Effect of ASS1 suppression on the experimental metastasis of human gastric cancer cell clones *in vivo*. NOD/SCID mice were intrasplenically injected with VC or RNAi-1 MKN45 cancer cell clones on day 14. The histologic images show a part of a mouse liver lobule bearing tumor nodules near the central vein (a-f) and a part of a spleen bearing tumor nodules (g-l). Tumor cells are indicated by arrows. H&E staining was performed to determine the distribution of tumor cells from the VC (a-c, g-i) and RNAi-2 (d-f, j-l) gastric cancer cell clones in paraffin-embedded specimens of the liver and spleen. The boxed areas in (a), (d), (g) and (j) are shown at higher magnification in (b), (e), (h) and (k), respectively (100x). The boxed areas in (b), (e), (h) and (k) are shown at a higher magnification in (c), (f), (i) and (l), respectively (400x). CV: central vein.

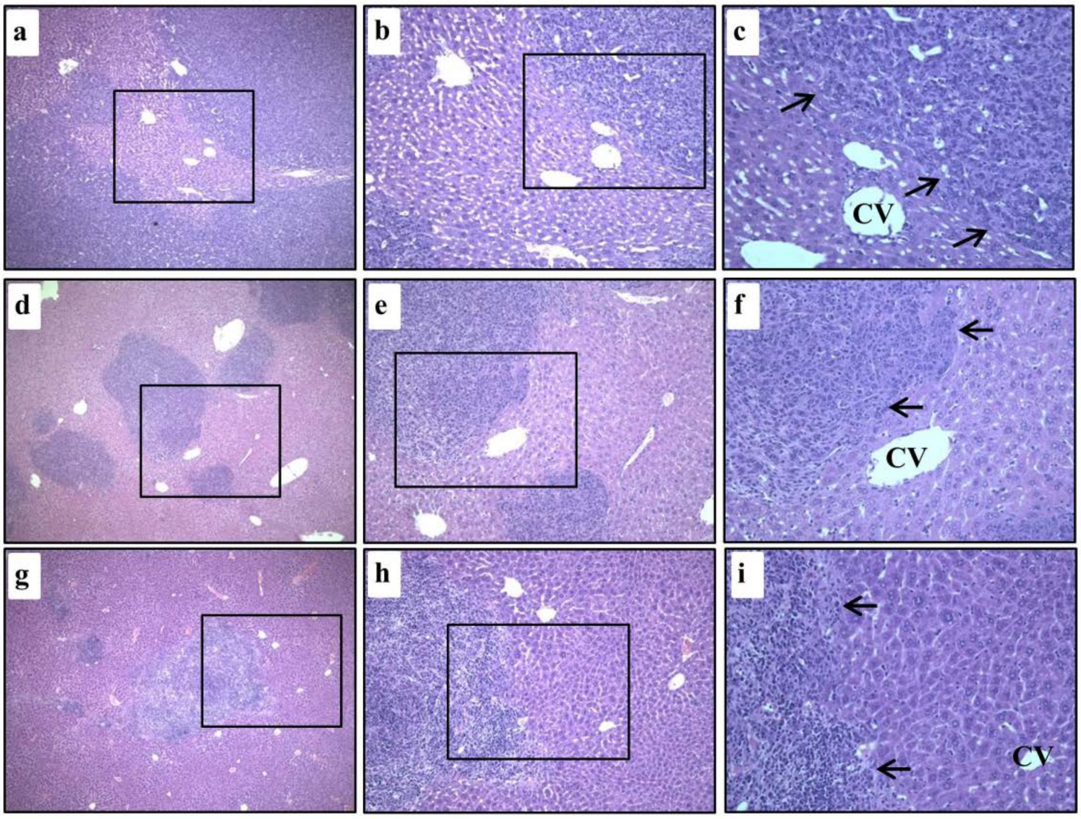

Supplementary Figure S6. Effect of Ass1 suppression on the experimental metastasis of murine gastric cancer cell clones *in vivo*. Mice were intrasplenically injected with 3IB2 VC, RNAi-1, or RNAi-2 cancer cell clones on day 11. The histologic images show a part of a mouse liver lobule bearing tumor nodules near the central vein. Tumor cells are indicated by arrows. H&E staining was performed to determine the distribution of tumor cells from the VC (a-c), RNAi-1 (d-f), and RNAi-2 (g-i) 3IB2 gastric cancer cell clones in paraffin-embedded specimens. The boxed areas in (a), (d) and (g) are shown at a higher magnification in (b), (e) and (h), respectively (100x). The boxed areas in (b), (e) and (h) are shown at a higher magnification in (c), (f) and (i), respectively (400x). CV: central vein.

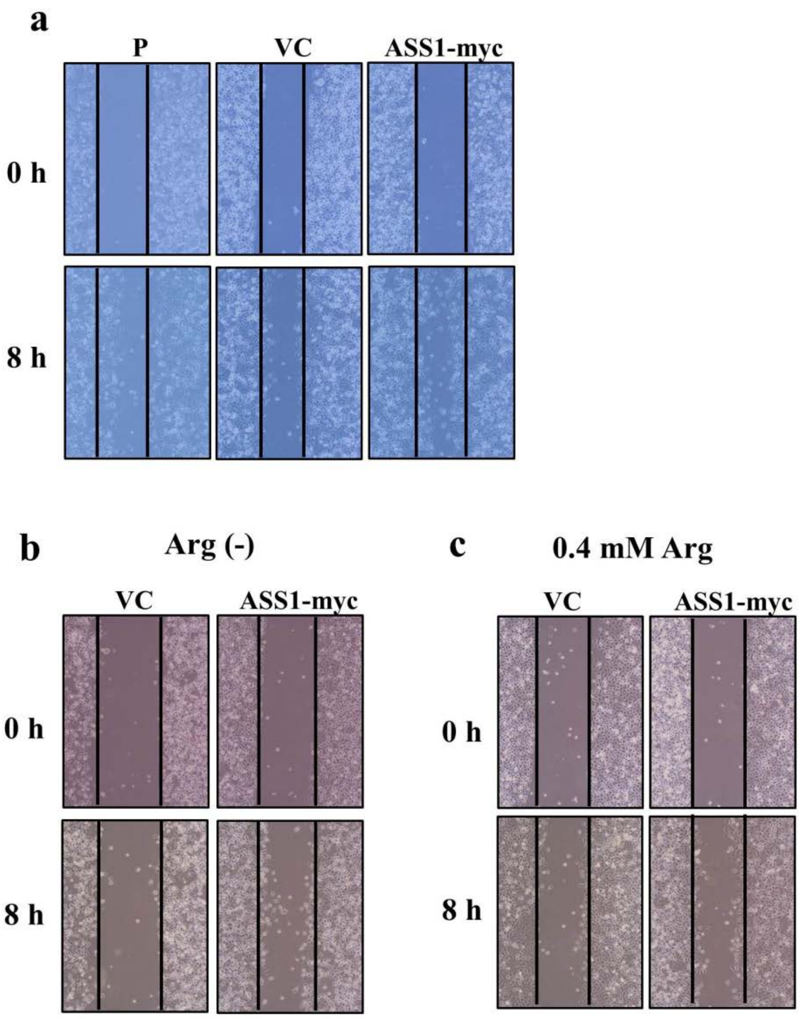

Supplementary Figure S7. ASS1 overexpression in AGS cell clones enhanced cell migration *in vitro*. (a) An ASS1-overexpressing cell clone was evaluated by the wound-healing assay. Cell motility toward the gap area was photographed after 8 h. Representative micrographs of one of the individual experiments at the time points 0 and 8 h are shown. Two different medium conditions were used as follows: (b) Arg (-): arginine-free; and (c) 0.4 mM arginine. All data were obtained from three independent experiments. P: parental cells; VC: vector control; ASS1-myc: ASS1- and myc-overexpressing. NS, not significant, *P < 0.05, **P < 0.001, ***P < 0.0001.

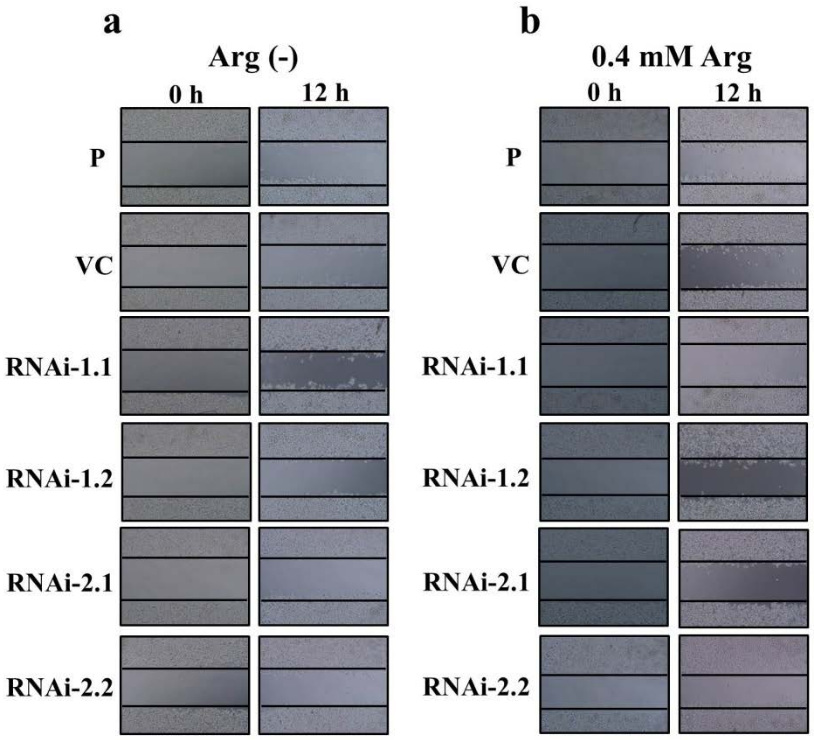

Supplementary Figure S8. Arginine deprivation affected cell migration in human MKN45 cell clones. Representative micrographs of one of the individual experiments at time points 0 and 12 h are shown. Cell motility toward the gap area was photographed after 12 h. Two different medium conditions were used as follows: (a) Arg (-): arginine-free; and (b) 0.4 mM arginine. P: parental cells; VC: vector control; and RNAi-1 and RNAi-2: ASS1-specific shRNAs 1 and 2, respectively.

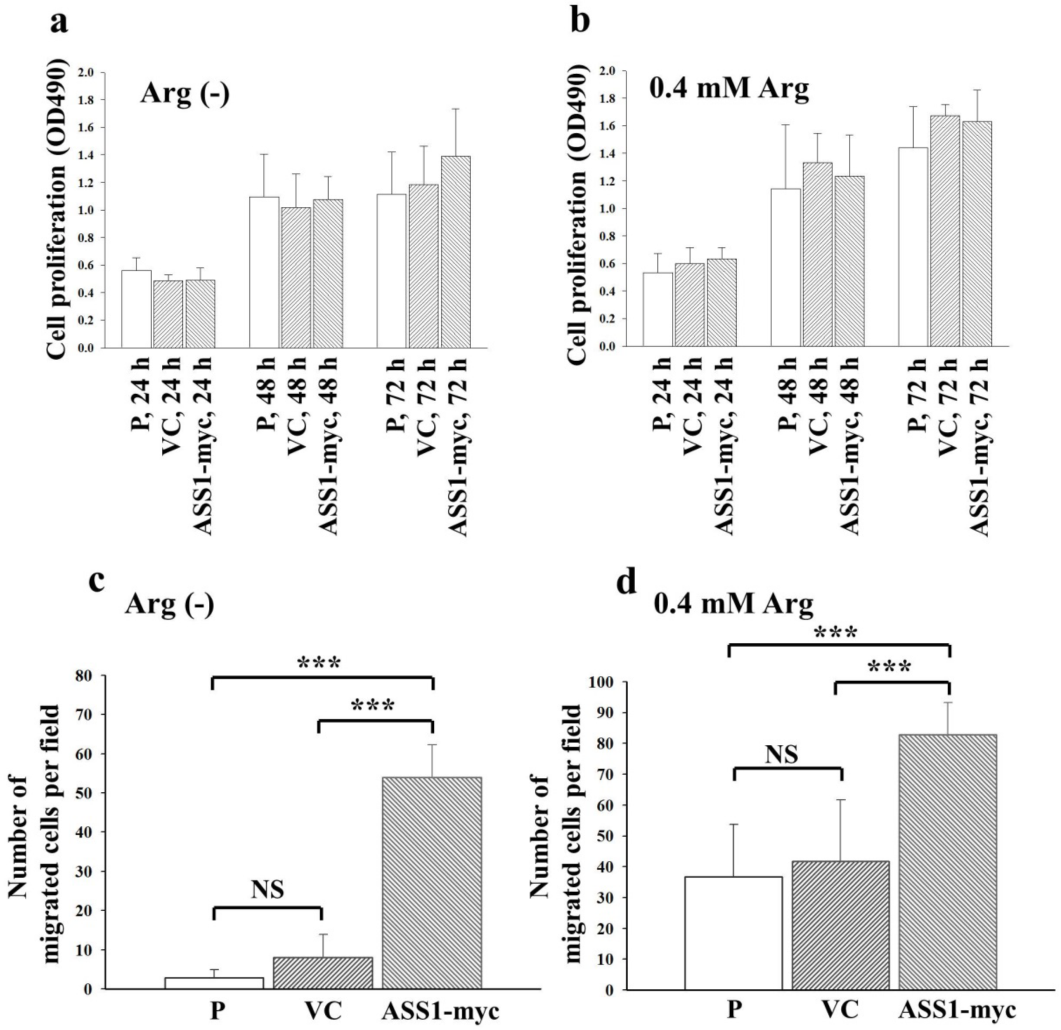

Supplementary Figure S9. The effect of arginine depletion on the growth and migration of human AGS gastric cancer cells. ASS1-overexpressing cell clones were grown in culture medium containing 5% FCS. Two different medium conditions were used. The cells were cultured for 24, 48, or 72 h. (a&b) Cell proliferation. (c&d) Wound healing. The results are expressed as the mean ± s.d. of three independent experiments. P: parental cells; VC: vector control; ASS1-myc: ASS1- and myc-overexpressing; Arg (-): arginine free. NS, not significant, ***P < 0.0001.

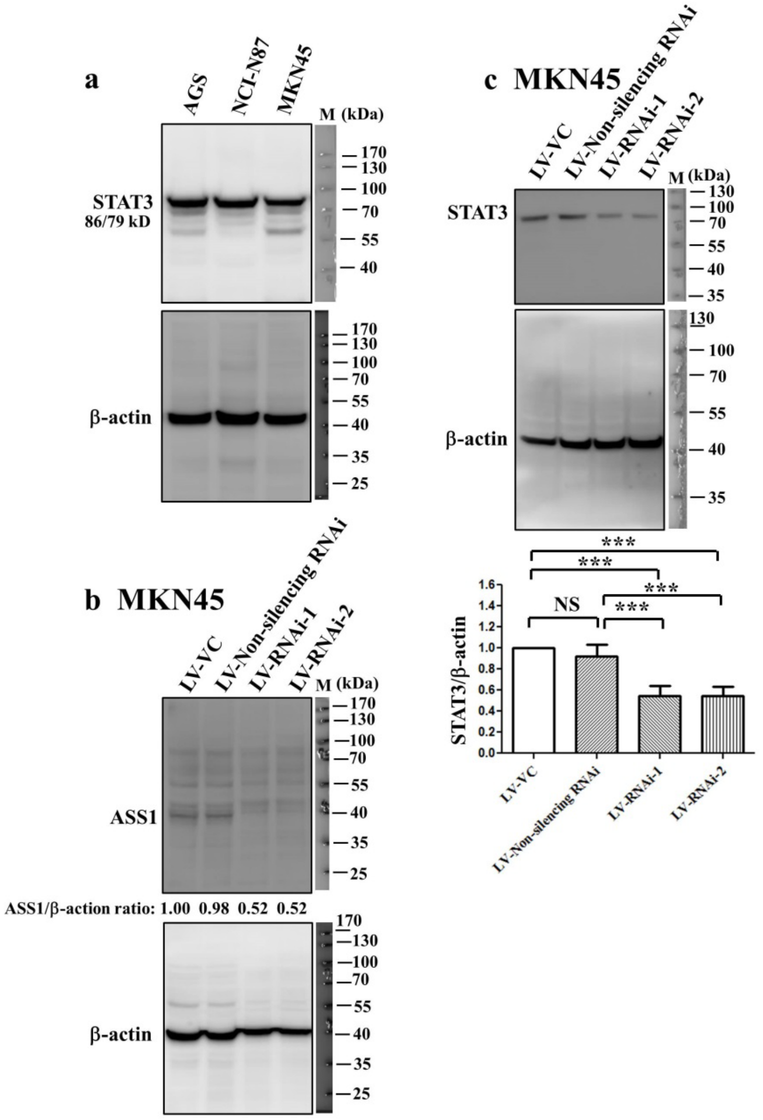

Supplementary Figure S10. (a) The protein expression levels of ASS1 and STAT3 were determined in AGS, NCI-N87, and MKN45 cells cultured in serum-deprived medium. (b) Lentivirus-mediated shRNA targeting ASS1 inhibited ASS1 protein expression. Transient transfection of either ASS1 shRNA specifically reduced STAT3 expression in MKN45 gastric cells. The bars represent the mean ± s.d. The results were obtained from three independent experiments. LV: lentivirus; P: parental cells; VC: vector control; RNAi-1 and RNAi-2: ASS1-specific shRNAs 1 and 2, respectively. NS, not significant, ***P < 0.0001.

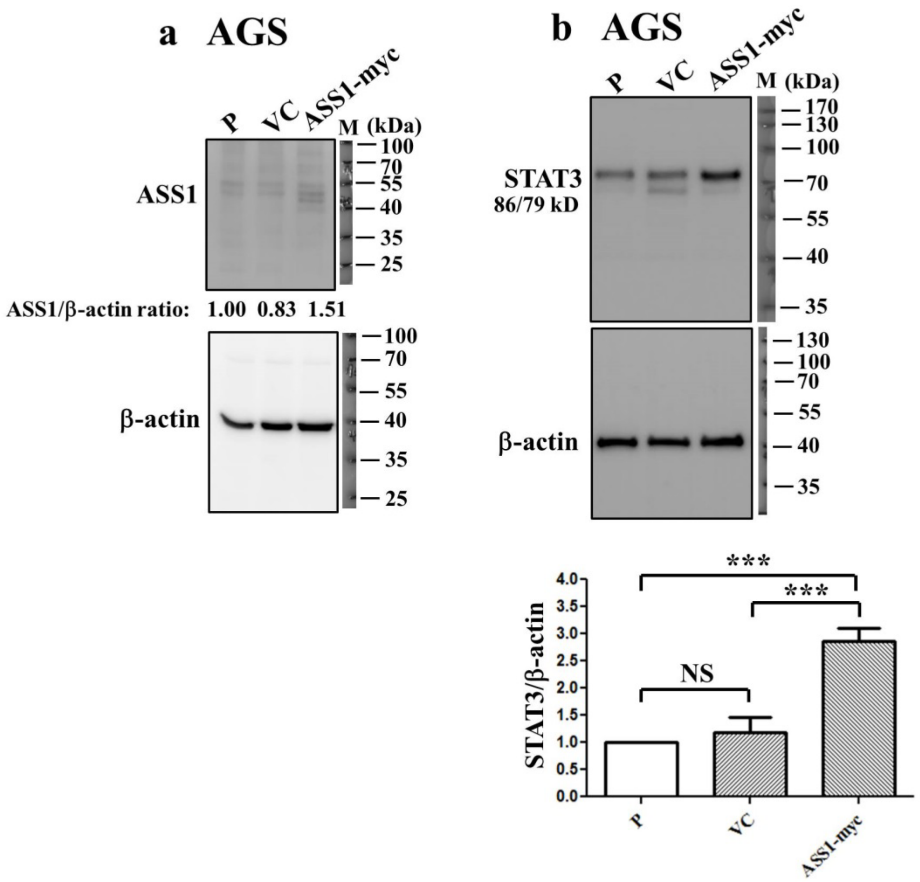

Supplementary Figure S11. ASS1 overexpression increased ASS1 protein expression in AGS cell clones. The upregulation of ASS1 (a) increased STAT3 protein expression (b) in AGS cells. The data represent the mean ± s.d. of three independent experiments. STAT3 isoform expression appears as STAT3α (86 kDa) and STAT3β (79 kDa). Cell lysates were immunoblotted using anti-ASS1, anti-STAT3 and anti-β-actin antibodies. P: parental cells; VC: vector control; ASS1-myc: ASS1- and myc-overexpressing; and NS, not significant, ***$P < 0.0001$.

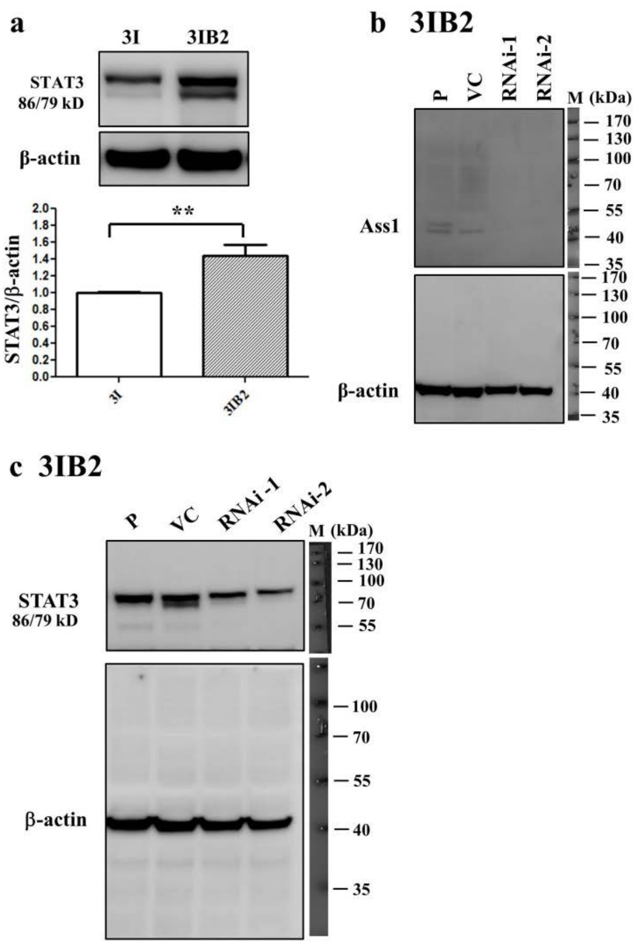

Supplementary Figure S12. The knockdown of Ass1 expression decreased STAT3 protein expression in murine gastric cancer cell lines. (a) The protein expression levels of Ass1 and STAT3 in 3I and 3IB2 cells were detected in cell clones cultured in serum-deprived medium. A significant increase in the STAT3 protein levels was observed in the 3IB2 compared with the 3I cells (**$P < 0.001$). The data represent the mean ± s.d. of three independent experiments. (b&c) Cell lysates were immunoblotted using anti-ASS1, anti-STAT3 and anti-β-actin antibodies. These cropped blots are shown in Fig. 6b. P: parental cells; VC: vector control; RNAi-1 and RNAi-2: Ass1-specific shRNAs 1 and 2, respectively. STAT3 isoform expression appears as STAT3α (86 kDa) and STAT3β (79 kDa).

1 Shan, Y. S. *et al.* Establishment of an orthotopic transplantable gastric cancer animal model for studying the immunological effects of new cancer therapeutic modules. *Mol. Carcinog.* **50**, 739-750 (2011).

2 Guerreiro, J. R. *et al.* Argininosuccinate synthetase is a functional target for a snake venom anti-hypertensive peptide - role in arginine and nitric oxide production. *J. Biol. Chem.* **284**, 20022-20033 (2009).
